# Do Treatment Choices by Artificial Intelligence Correspond to Reality? Retrospective Comparative Research with Necrotizing Enterocolitis as a Use Case

**DOI:** 10.1177/0272989X251324530

**Published:** 2025-03-12

**Authors:** Rosa Verhoeven, Stella Mulia, Elisabeth M. W. Kooi, Jan B. F. Hulscher

**Affiliations:** Department of Surgery, Division of Pediatric Surgery, University of Groningen, University Medical Center Groningen, Groningen, The Netherlands; Department of Neonatology, Beatrix Children’s Hospital, University of Groningen, University Medical Center Groningen, Groningen, The Netherlands; Councyl B.V., Delft, The Netherlands; Department of Neonatology, Beatrix Children’s Hospital, University of Groningen, University Medical Center Groningen, Groningen, The Netherlands; Department of Surgery, Division of Pediatric Surgery, University of Groningen, University Medical Center Groningen, Groningen, The Netherlands

**Keywords:** necrotizing enterocolitis, artificial intelligence, decision support, decision making, end-of-life decision, comfort care, retrospective comparison

## Abstract

**Background:**

In cases of surgical necrotizing enterocolitis (NEC), the choice between laparotomy (LAP) or comfort care (CC) presents a complex, ethical dilemma. A behavioral artificial intelligence technology (BAIT) decision aid was trained on expert knowledge, providing an output as “*x* percentage of experts advise laparotomy for this patient.” This retrospective study aims to compare this output to clinical practice.

**Design:**

Variables required for the decision aid were collected of preterm patients with NEC for whom the decision of LAP or CC had been made. These data were used in 2 BAIT model versions: one center specific, built on the input of experts from the same center as the patients, and a nationwide version, incorporating the input of additional experts. The Mann–Whitney *U* test compared the model output for the 2 groups (LAP/CC). In addition, model output was classified as advice for LAP or CC, after which the chi-square test assessed correspondence with observed decisions.

**Results:**

Forty patients were included in the study (20 LAP). Model output (*x* percentage of experts advising LAP) was higher in the LAP group than in the CC group (median 95.1% v. 46.1% in the center-specific version and 97.3% v. 67.5% in the nationwide version, both *P* < 0.001). With an accuracy of 85.0% by the center-specific and 80.0% by the nationwide version, both showed significant correspondence with observed decisions (*P* < 0.001).

**Limitations:**

We are merely examining a proof of concept of the decision aid using a small number of participants from 1 center.

**Conclusions:**

This retrospective study demonstrates that treatment choices by artificial intelligence align with clinical practice in at least 80% of cases.

**Implications:**

Following prospective validation and ongoing refinements, the decision aid may offer valuable support to practitioners in future NEC cases.

**Highlights:**

- This study assesses the output of behavioral artificial intelligence technology in deciding between laparotomy and comfort care in surgical necrotizing enterocolitis.
- The model output aligns with clinical practice in at least 80% of patient cases.
- Following prospective validation, the decision aid may offer valuable support to physicians working at the neonatal intensive care unit.

## Introduction

Doctors managing patients with necrotizing enterocolitis (NEC), a devastating disease affecting the intestines of predominantly very premature babies,^1–3^ frequently encounter a difficult decision. In cases of surgical NEC, defined by pneumoperitoneum and/or clinical deterioration despite maximal medical therapy,^4^ a decision must be made between 2 distinct paths: pursuing laparotomy (LAP) or diverting to palliative care (i.e., comfort care [CC]) instead. This is one of the hardest moral decisions in health care and involves an estimation of the patient’s chance of survival and future quality of life.^5^ Perioperative mortality rates can reach 50%, and survivors may face long-term complications such as morbidity secondary to short bowel syndrome or neurodevelopmental delay in more than 57%.^6–8^ Because of these complications, the doctors may be confronted with the dilemma as to whether LAP is still in the best interest of the patient.^5,9^

This dilemma is usually discussed in multidisciplinary team meetings, often attended by the neonatologist, pediatric surgeon, and the pediatric anesthesiologist. These physicians must first determine whether the treatment path can be considered ethically impermissible, ethically permissible, or ethically obligatory.^10^ In case both LAP and CC can be considered ethically permissible, the options have to be discussed with the parents. However, determining the advisability of each treatment option can be challenging.

Artificial intelligence has the potential to support physicians in these types of medical decision making, although machine learning systems are often considered opaque and require vast amounts of historical data.^11–13^ However, population data on the prognosis of surgical NEC are limited; for example, only 10 to 20 cases occur annually at the University Medical Center Groningen (UMCG), a Dutch tertiary health hospital that has a catchment area of 3.5 million people. Furthermore, evaluating decision quality is challenging due to the lack of objective measures. Therefore, this approach might not be suitable for the issue at hand. Traditional rule-based expert systems, on the other hand, are often incapable of capturing the moral complexity and subtleties involved in such decisions.^14^ Hence, recently, an alternative decision support approach based on behavioral artificial intelligence technology (BAIT) was presented, which uses choice analysis techniques to map specialized expertise into a decision support model.^15^

BAIT is a novel technology that allows for developing decision aids using the knowledge of experts. In a previous study, we asked experts throughout the Netherlands to perform a choice experiment in which they had to choose between LAP or CC in several hypothetical situations of patients with surgical NEC.^16^ The situations in the experiment were created by combining values of predetermined decision criteria (Table 1) in such a way that they resulted in difficult tradeoffs. By analyzing the decisions made by the experts, BAIT allows for identifying the weights of the decision criteria and exposing hidden bias and heterogeneity.^17^

**Table 1:** Predetermined Variables Required for the BAIT Model, Including Possible Values and the Variables’ Relative Importances in the Center-Specific and the Nationwide BAIT Model^a^

		Relative Importance	
BAIT Variable	Possible Values	Center-Specific Model (%)	Nationwide Model (%)
Sex	[boy, girl]	2.05	1.21
Gestational age	Gestational age in weeks	12.97	9.76
Birth weight	Birth weight in grams	15.52	12.68
Perinatal asphyxia	[yes, dubious, no]	5.87	4.63
Congenital comorbidities	[present with high impact, present with minor impact, absent]	7.65	10.09
Clinical course pre-NEC	[serious complications, minor complications, no complications]	4.71	4.36
Postnatal age	Postnatal age in days	4.03	3.14
Growth since birth	[weak, intermediate, good]	3.09	1.23
Cerebral ultrasound	[poor prognosis, intermediate prognosis, good prognosis]	16.17	20.46
Lung function	[weak, intermediate, good]	3.31	5.24
Hemodynamics	[unstable despite maximum inotropic support, stable with inotropic support, stable without inotropic support]	9.44	11.14
Cerebral oxygenation (measured with near-infrared spectroscopy)	Oxygenation in percentage	4.79	4.52
Parental preferences	[in favor of comfort care, doubtful about surgery, in favor of surgery]	9.50	9.41
Estimated parental capacities (for future care for NEC survivor)	[weak, intermediate, good]	0.90	2.13

a Relative importance provides an approximation of the contribution of the criterion toward the model output in respect to other criteria. It is calculated as the percentage contribution of each criterion to the total theoretical maximum effect summed over all criteria. BAIT, behavioral artificial intelligence technology; NEC, necrotizing enterocolitis.

**Figure 1:**
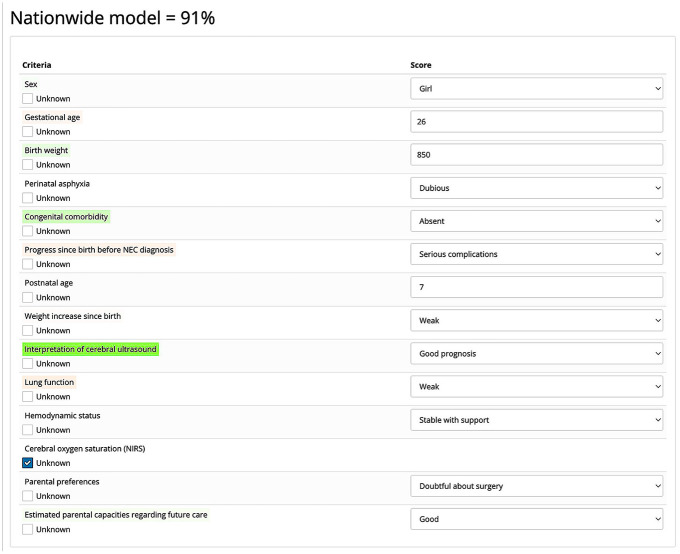
Example of the nationwide choice model’s output. The probability that a randomly sampled expert from the Dutch group of experts who performed the choice experiment would recommend performing surgery on a patient with this profile equals 91%. Color coding indicates the positive or negative influence of the variables on this output. Green indicates a positive effect, red indicates a negative effect, and the intensity of the color represents the weight of that effect. Unknown variables can be left blank by selecting the “Unknown” checkmark. They are treated neutrally.

Using this knowledge, a final model can be constructed that takes the values of the decision criteria of a new patient as input and provides output formulated as: “*x* percentage of experts advise laparotomy for this patient” (Figure 1). Two such models were developed for the decision between LAP and CC in surgical NEC: 1 center-specific version based on the decisions made by 17 experts from the UMCG who participated in the choice experiment, and 1 nationwide version based on the decisions of all 62 national experts who participated in the experiment.^16^ Among these experts were E.M.W.K. and J.B.F.H. as well as other neonatologists and pediatric surgeons.^16^ The aim of the current study is to assess the practicality of the constructed decision aids as a proof of concept and to evaluate their potential for use in clinical practice.

The assessment of the models will involve retrospectively comparing their output with the observed choices in patients with surgical NEC at the UMCG, upon whose experts the center-specific model is constructed. As the 2 models are slightly different in the weights of the factors, we expect to see different outputs. We might observe greater correspondence between practice and output from the center-specific model than from the nationwide model. Finally, if discrepancies exist between models’ output and practice, more in-depth explorative research will be performed to identify and learn from the deviating cases.

If there is a substantial correspondence between the two, we can infer that the models reflect medical decision making. In that case, we can use the model to support more unified decision making among health care professionals and to guide the identification and advisability of the different treatment options as well as contributing factors. This knowledge can be shared during parental counseling, thereby empowering the parents and optimizing shared decision making.

Although the examined models serve as a starting point, the ultimate goal is to develop a comprehensive international model for treatment of surgical NEC. In addition, the findings of this study could serve as proof of concept for the effective utilization of BAIT in similar morally complex health care decisions.

## Methods

### General Setup

To compare the model output to practice retrospectively, the required variables (Table 1) of patients with NEC for whom the decision of LAP versus CC had been made were entered as input into the models (like in Figure 1). After this, for both models, an output was provided in the form of a probability statement (*x* percentage of experts in support of LAP) for each patient along with the influence that the variables have on this output. The average model output was compared between the group of patients who received LAP and the group of patients for whom CC was chosen. Next to this, for both the center-specific and nationwide model, an output cutoff value was determined (below cutoff is classified as advice for CC, equal to or greater is classified as advice for LAP) for the models to correspond with reality as closely as possible. The models’ accuracy at this cutoff value functions as a surrogate for their potential support in future NEC cases. Finally, possible discrepancies between model output and clinical practice (using the determined cutoff value as reference point) were explored by paying additional attention to these patients and looking at the values of the variables with the highest relative importance. The study was approved by the Ethical Board of the UMCG. Financial support was provided by a grant from the For Wis(h)dom Foundation, which enabled the entire study to be conducted.

### Patient Selection

**Figure 2:**
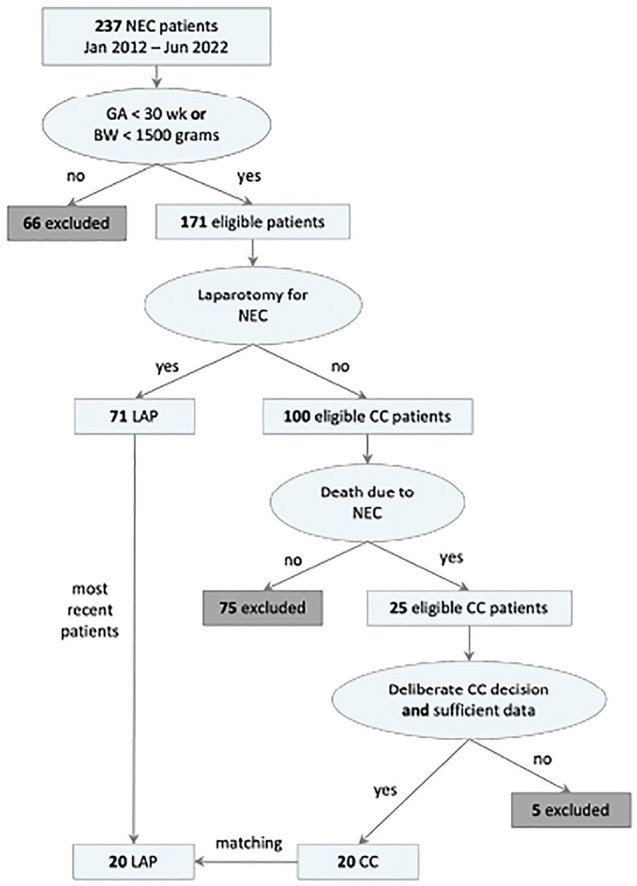
Patient selection process.Only patients with gestational age (GA) less than 30 wk or with birth weight (BW) less than 1,500 g were included. If they received laparotomy for necrotizing enterocolitis (NEC), they could be included in the laparotomy (LAP) group. If they died as a result of NEC without receiving laparotomy, they could be included in the comfort care (CC) group.

Figure 2 shows an overview of the first part of the selection process. Patients were selected from a prospectively collected database of patients with NEC (Bell’s stage ≥2a, i.e., definite NEC^18^) who were admitted to the neonatal intensive care unit of the UMCG between January 2012 and June 2022. In this time frame, no substantial changes have been made in NEC treatment procedures. Only patients with a birth weight below 1,500 g or a gestational age less than 30 wk were included, as these are highest-risk patients for whom the decision aid would be most relevant. Next, the patients for whom the decision of LAP versus CC has been made were determined. Since the decisions made in the multidisciplinary team meetings were not recorded explicitly in the database, 2 inclusion criteria were used. First, patients who had received LAP were included, in which case the decision had clearly been made. Second, patients who had died from NEC without receiving LAP were included for review, as this indicates that the decision of CC might have been made. Their patient files were examined more thoroughly. If a patient died regardless of therapeutic conversative treatment (nonsurgical interventions aimed at managing the condition), if there were insufficient data to generate a reliable model output, or if it was not convincingly established that the patient’s death resulted specifically from NEC rather than other causes, the patient was excluded. Consequently, the final group of patients consisted solely of those at Bell’s stage 3 (of 3, i.e., advanced NEC^18^).

To ensure a balanced comparison between patients who underwent LAP and patients who received CC, we matched the number of patients in the smallest group by selecting an equal number of recent cases from the larger group, thereby achieving an equal distribution for analysis. With an expected accuracy of 0.85 and an equally distributed sample of 40 patients in total, the 95% confidence interval lies between 0.74 and 0.96,^19^ which would be sufficient to determine the models’ performance.

### Procedure

Two senior neonatologists and 2 senior pediatric surgeons working at the UMCG offered to help review patient data. The selected 40 patients were randomly divided among these clinicians for review. By e-mail, the clinicians were requested to determine the patients’ required variables from the patient records and to document them along with a patient study ID in REDCap, the electronic data-capturing tool of the UMCG. The researcher could not influence or adapt their responses. The physicians were allowed to pause and resume the documentation at their own convenience.

The physicians were informed that some variables required interpretation, and they were asked to select the most representative value of the patient’s situation at the moment that the decision of LAP versus CC was made. In addition, the physicians were instructed to leave variables empty in case it was not possible to determine a value for it, for example, if no relevant data had been documented. These variables are treated as neutral (i.e., not having a positive or negative effect on the final output). Finally, the physicians were asked to fill in the data as objectively as possible, without considering the patient’s future course.

Once the required variables were obtained for all patients, the data were uploaded onto the BAIT software, which returned the probability statements and influence of the different variables.

### Analysis

All of the statistical analyses were performed in R.^20^ Patient characteristics are displayed as percentages for categorical data and mean with standard deviation or median with interquartile range (IQR) for continuous data, as appropriate. Categorical data were compared between the 2 groups (LAP and CC) with chi-square (χ^2^) tests, normally distributed continuous variables with the Student’s *t* test, and nonnormally distributed continuous variables with the Mann–Whitney *U* test. Model output between LAP and CC was compared with the Mann–Whitney *U* test for both the nationwide and center-specific model separately. In addition, the model output for the LAP group was compared between the nationwide and center-specific model. The same was done for the CC group.

By calculating the sensitivity, specificity, and accuracy of the 2 models for cutoff values between 1% and 99%, we determined at which cutoff value the model outputs matched observed choices in clinical practice as closely as possible. By increasing the cutoff value, more cases are classified as advice for CC. This means that specificity (percentage of patients who were classified as advice for LAP and truly received LAP) increases, while sensitivity (percentage of patients who received LAP and were also classified as advice for LAP) decreases. The situation might occur where the highest accuracy is obtained at a cutoff value at which preference is given to either sensitivity or specificity. Although some might argue that we should prioritize sensitivity to be sure of operating on babies with a potentially good prognosis, others might say that it would be better to prioritize specificity and focus on the infants’ quality of life. Given that we wanted to prevent favoring either one of these viewpoints, the accuracy at the cutoff value at which sensitivity and specificity cross was taken as a measure to see how well the models represent reality. Then we took the models’ decisions at this cutoff value and used the chi-square test to assess the correspondence between the categorized model outputs and the observed choices.

Finally, for the explorative analysis of the differently classified patients, we examined cerebral ultrasound, birth weight, hemodynamics, gestational age, congenital comorbidity, and parental preferences, which have been shown to be important factors to consider when making the decision of LAP versus CC.^16^ If this did not explain the choices, an additional review of the patient’s charts was performed.

## Results

A final selection of 20 patients in the CC group was established, compared with 71 patients in the LAP group. For this reason, we matched the number of participants by including only the 20 most recent patients who received LAP. We did not observe clinically or statistically significant differences in sex (65.0% and 60.0%, respectively) or age at NEC onset (median 10.0 and 8.0 days, respectively) between the LAP and CC groups. For patients who received CC, we did find clinically and statistically lower gestational age (median 25+1 wk v. 27+3 wk for LAP, *P* < 0.001) and lower birth weight (median 753 g v. 963 g for LAP, *P* < 0.001) (Table 2).

**Table 2:** Characteristics of the Selected Group of NEC Patients^a^

Characteristic	All (*N* = 40)	LAP (*n* = 20)	CC (*n* = 20)	Difference LAP/CC
Male (%)	25 (63)	13 (65)	12 (60)	*P* = 0.74
Median gestational age in weeks + days (IQR)	26 + 0 (25 + 0 - 27 + 5)	27 + 3 (26 + 1 - 28 + 5)	25 + 1 (24 + 5 - 25 + 6)	*P* < 0.001
Mean birth weight in grams (*s*)	858 (205)	963 (211)	753 (136)	*P* < 0.001
Median age in days at NEC onset (IQR)	9.5 (6.0–12.5)	10.0 (6.75–15.0)	8.0 (6.0–11.3)	*P* = 0.27

CC, comfort care; IQR, interquartile range; LAP, laparotomy; NEC, necrotizing enterocolitis; *s*, standard deviation.

a Difference between the LAP and CC group as determined by chi-square test for categorical data, *t* test for normally distributed continuous data, Mann–Whitney–Wilcoxon test for nonnormally distributed continuous data.

### Model Output Comparison

**Figure 3:**
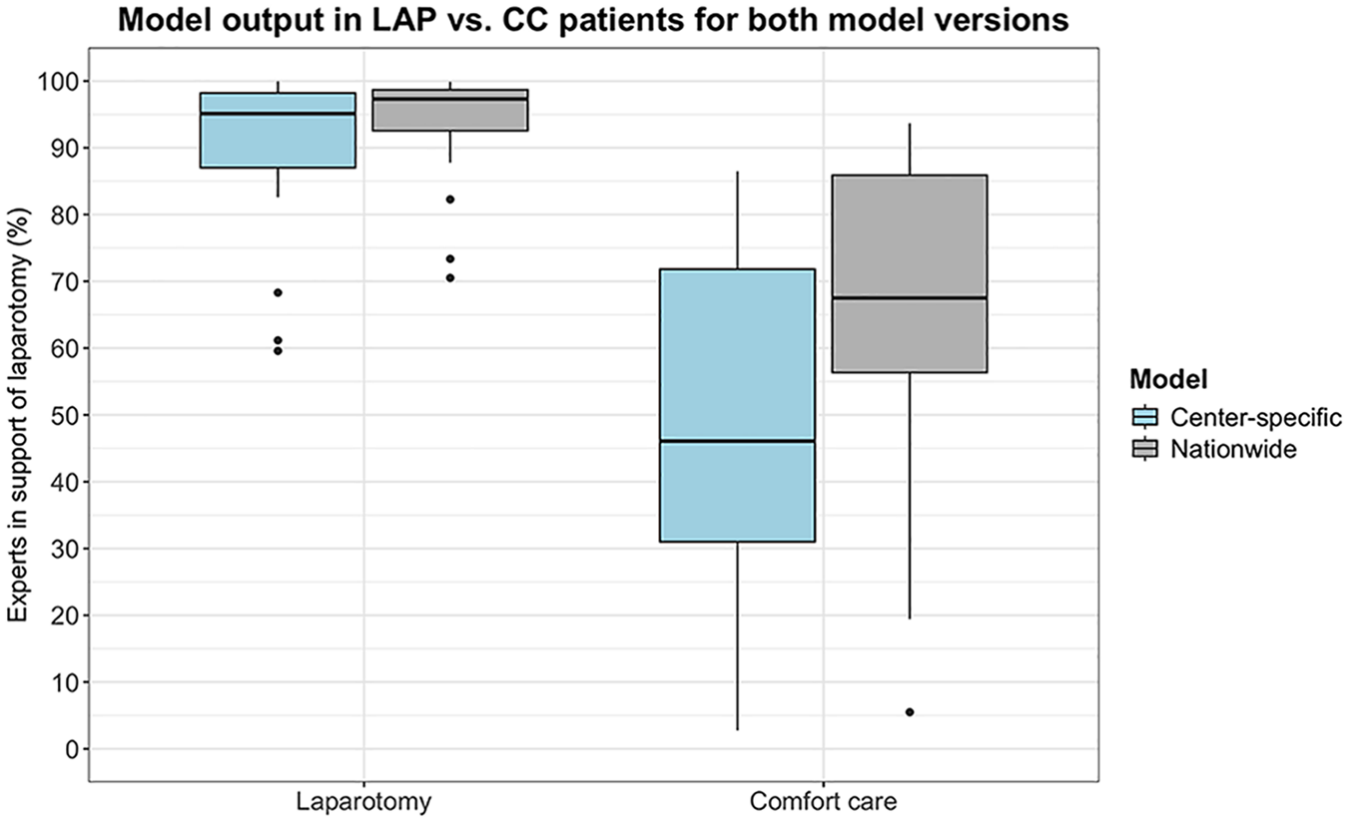
Model output (percentage experts in support of laparotomy) for the 2 patient groups (laparotomy [LAP] and comfort care [CC]) by both the center-specific model and the nationwide model. The individual dots represent model outputs that fall outside the interquartile range.

For both models, a difference in model output can be observed between LAP and CC patients (Figure 3). The center-specific model’s output (percentage experts in support of LAP) is higher for the patients who received LAP (median 95.1%, IQR = 87.0–98.2) than for those who received CC (median 46.1%, IQR = 31.0–71.8) (*P* < 0.001). Similarly, the nationwide model’s output (percentage experts in support of LAP) is also higher for the patients who received LAP (median 97.3%, IQR = 92.6–98.7) than for the patients who received CC (median 67.5%, IQR = 56.3–85.9) (*P* < 0.001).

We found no significant difference in output between the center-specific model and the nationwide model for the LAP group (median 95.1%, IQR = 87.0–98.2 and median 97.3%, IQR = 92.6–98.7, respectively) (*P* = 0.27). However, we did find a higher model output (percentage experts in support of LAP) in patients who received CC by the nationwide model (median 67.5%, IQR = 56.3–85.9) than by the center-specific model (median 46.1%, IQR = 31.0–71.8) (*P* = 0.040).

**Figure 4:**
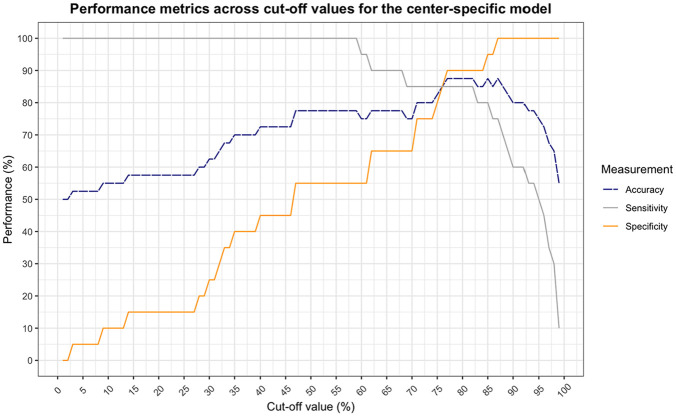
Performance of the center-specific behavioral artificial intelligence technology (BAIT) model as a function of cutoff value (less than the cutoff is classified as advice for comfort care [CC], equal to or greater than is classified as advice for laparotomy [LAP]). Sensitivity and specificity cross at a cutoff value of 76%, at which the accuracy is 85.0%.

**Figure 5:**
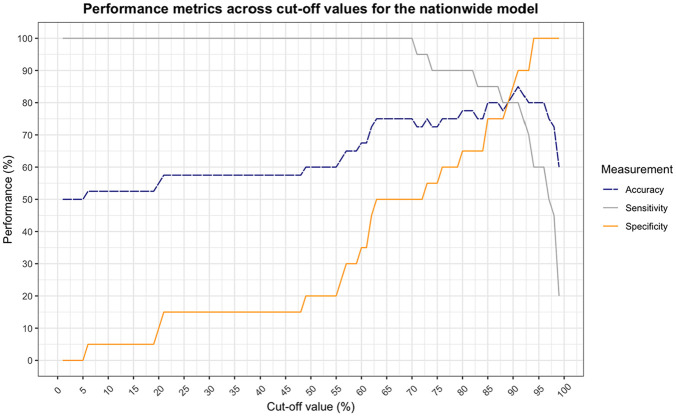
Performance of the nationwide behavioral artificial intelligence technology (BAIT) model as a function of cutoff value (less than the cutoff is classified as advice for comfort care [CC], equal to or greater than is classified as advice for laparotomy [LAP]). Sensitivity and specificity cross at a cutoff value of 89%, at which the accuracy is 80.0%.

The center-specific model shows a balance between sensitivity and specificity at a cutoff value of 76% (Figure 4). At this point, the model obtains an accuracy of 85%. This is a significant correspondence with observed choices in clinical practice (χ^2^[1] = 19.6, *P* < 0.001). The nationwide model shows a slightly lower but still significant correspondence with an accuracy of 80% at a cutoff value of 89% (χ^2^[1] = 14.4, *P* < 0.001) (Figure 5).

### Discrepancies between Model Output and Practice

At a cutoff value of 76% for the center-specific model and 89% for the nationwide model, 9 patients in total were classified as advice for CC, whereas they received LAP or vice versa (Table 3): 6 patients were classified differently by the center-specific model and 8 by the nationwide model. Five patients were classified differently by both models.

**Table 3:** Data of Differently Classified Patients^a^

Patient	Observed Choice	Center-Specific Model (Classification)	Nationwide Model (Classification)	Gestational Age (wk)	Birth Weight (g)	Cerebral Ultrasound	Hemodynamics	Congenital Comorbidities	Parental Preferences
1	LAP	61.2% (CC)	73.4% (CC)	24–25	500–750	Good	Stable with support	Present with high impact	LAP
2	LAP	59.6% (CC)	70.5% (CC)	28–29	750–1,000	Intermediate	Stable with support	Present with high impact	LAP
3	LAP	68.3% (CC)	82.3% (CC)	24–25	500–750	Intermediate	Stable with support	Absent	LAP
4	LAP	82.6% (LAP)	87.8% (CC)	26–27	1,000–1,250	Intermediate	Stable without support	Absent	Doubtful
5	CC	74.8% (CC)	90.6% (LAP)	24–25	500–750	Good	Stable with support	Absent	Unknown
6	CC	70.8% (CC)	89.6% (LAP)	24–25	750–1,000	Good	Stable with support	Absent	CC
7	CC	84.0% (LAP)	93.1% (LAP)	24–25	750–1,000	Good	Stable without support	Absent	CC
8	CC	76.8% (LAP)	88.8% (CC)	24–25	750–1,000	Good	Stable with support	Absent	CC
9	CC	86.5% (LAP)	93.7% (LAP)	24–25	<500	Good	Stable with support	Absent	LAP

CC, comfort care; LAP, laparotomy.

a The table shows the actual treatment that has been chosen for the patient along with model outputs (and the classification based on the cutoff values of 76% and 89% for the center-specific and nationwide model, respectively). The values of the most informative behavioral artificial intelligence technology (BAIT) variables as reported by the physicians are also provided. Gestational age and birth weight have been categorized to provide anonymity.

Three patients are classified as advice for CC by both models, although they received LAP in practice. The models showed a relatively low model output, mainly due to low gestational age and/or birth weight and the presence of congenital comorbidities, in combination with an intermediate prognosis based on the cerebral ultrasound and the requirement of inotropic support for stable hemodynamics. However, in all 3 cases, the parents were in favor of surgery. Further review of the charts showed that 2 of these 3 patients died after surgery. The 1 survivor showed normal cognitive development at the 2-y follow-up, with only minor behavioral difficulties. The patient who received LAP but was classified as advise for CC by the nationwide model, but not by the center-specific model, also died.

Five patients were classified as advice for LAP by at least 1 of the models, whereas they received CC in practice. The model output was relatively high due to the lack of congenital comorbidities, good prognosis based on cerebral ultrasound, and stable hemodynamics, among others. However, in 3 of the cases, the parents were in favor of CC. For 1 patient, the parents’ preferences were not clear and left as unknown in the model. These parents indicated that they did not want their child to suffer, which the doctors suspected would happen disproportionally if they were to continue with surgical treatment, as retrieved from the charts. Only 1 patient’s parents were in favor of LAP, but for this patient, the doctors decided not to perform surgery due to low gestational age, extremely low birth weight (<500 g), ongoing severe thrombopenia unresponsive to platelet transfusions, and severe ongoing sepsis regardless maximized broad spectrum antibiotics.

## Discussion

In this study, we investigated the correspondence between BAIT and practice in retrospectively selected cases from a single center to evaluate its suitability as decision support. The observed agreement between model output and reality suggests potential for the decision aid to be used in clinical practice.

As expected, we found that both the center-specific and nationwide model showed a significantly higher model output (percentage experts in support of LAP) for patients who received LAP compared with those who received CC. The center-specific model demonstrated a slightly greater correspondence with an accuracy of 85%, whereas the nationwide model achieved an accuracy of 80%. This higher correspondence is logically consistent, given that the patient data used in this study were sourced directly from the same center upon whose experts the center-specific model is built. Both models showed a significant correspondence with observed decisions.

An examination of the differently classified patients showed that in patients who received LAP, most important variable values were not outstanding (e.g., intermediate prognosis from cerebral ultrasound or presence of congenital comorbidities). Conversely, most patients who received CC had more positive values (e.g., good prognosis from cerebral ultrasound, no congenital comorbidities). In many of these cases, the parents’ preferences appeared to have a greater influence on the actual outcome than the models might have captured. This raises the question of what implications this holds for our models. On one hand, one could argue for not including the parents’ preferences into the models at all. We could build a model solely based on the patient’s clinical situation to avoid biased model outputs. This proposition gains further traction considering that of the 4 patients who underwent LAP while the model recommended CC, 3 unfortunately did not survive postsurgery. On the other hand, clinicians are often aware of the parents’ preferences, and as this knowledge may play a role in the decision process, we chose to include it as a factor at this stage already. The results indeed show that we factor in the parents’ preferences, even more so than we initially expected from the choice experiment. As we see it, the perhaps limited contribution of the variable in the model may now serve as a compromise between the 2 options of leaving out the variable or making its effect even stronger.

In other cases in which model output and practice did not align, the doctors’ opinions, which were based on factors outside the scope of the variables required for the models, seemed to predominate. Although we designed our model intentionally to encompass a set of relatively broadly interpretable factors that inherently encapsulate a wide range of implicit considerations, it is impossible to capture all of the clinical circumstances that might affect the final decision in a decision aid with BAIT. In 1 case in particular, we found that some variables and variable levels that are not explicitly captured in our model, such as extremely low birth weight, ongoing sepsis, and ongoing thrombopenia, contributed to the advice to divert to CC. Although one could argue that these factors are entailed in the variable “clinical course pre-NEC,” such extreme circumstances cannot be reflected by this variable because of the relatively low importance that it carries (Table 1). This aspect may need to be addressed in the design of future models, although it should be noted that this observation was seen in only 1 extreme case among 40 patients. Therefore, we still conclude that the models are able to make a solid approximation even with the limited number of variables that are currently included.

One of the greatest advantages of BAIT is that it has the ability to show which variables contributed to the final model output, allowing for a high degree of transparency in our decision making. It allows for a guided discussion about differences in conceptions between doctors about their patients. This can be useful in multidisciplinary team meetings about the child’s treatment to support more unified decision making but also to determine treatment advisability for the counseling conversation with parents. Thereby, it strongly contributes to the concept of shared decision making between doctors and parents. Although the current study has shown that the parents’ preferences might even play a larger role in the final decision than initially expected, some studies have shown that actual shared decision making is adopted to only a small extent in end-of-life medical decision-making conversations.^21^ This might have to do with the fact that one regularly implemented strategy for parental involvement by neonatologists is to just provide recommendations or single-option choices for the course of action.^22–24^ However, parents have indicated the desire to be presented with relevant knowledge for their child and the choice they have to make, which gives them the opportunity to ask questions and collaborate.^22,25^ By acknowledging the parents’ individuality by being able to report on the variables that influence their child’s outcome most, the parents can be empowered in the decision-making process as well.^26,27^ In future studies, we will also aim to develop a useful decision aid that can be used by parents directly.

One factor that might be considered a limitation of not only this study but also of the models themselves is the fact that variables can be interpreted differently across raters. In the current study, all patients were divided among the 4 experts, each having their own experience and biases that might influence their interpretation of the variables. Some variables are more prone to differences in interpretation than others, and some have a greater effect on the final model output. We have decided to develop our models like this for 2 reasons. First, we believe this is how we currently evaluate the patient’s situation in reality. Clinicians examine the clinical factors and assess their impact on the patient’s prognosis, acknowledging that it is not always possible to predict which specific values will correspond to a certain outcome. Second, and related to that, we are required to do it this way precisely because there is uncertainty. There are no definite predictive values for the patient’s prognosis or else we would not have to perform this experiment. Due to the lack of transparency that currently exists, we cannot define the variables perfectly. For that very reason, we are trying to set a first step toward understanding our decision making and unveiling the uncertainty that also exists in the interpretation of the patients’ situations. Once we have learned more about this, we can continue to define the criteria to achieve more consistent decision making.

In future research, we will investigate the differences in the way the variables are interpreted by different doctors in prospective cases. Asking the doctors to enter the variables of the patients individually also allows us to study the individual contribution of each doctor to the final decisions made in clinical practice, so we can learn more about the dynamics in the decision-making process between multiple doctors, which allows us to understand how the tool should be used optimally. In addition, the prospective setup allows us to eliminate bias in the interpretation of the variables that may have occurred as a result of the patients’ future courses and final outcomes being known already. This bias may have involved stronger positive interpretations for patients who received LAP and survived and more negative interpretations for patients who received CC or for those who received LAP and died after all. As we do not expect to see this behavior in doctors using the tool in the future, we may observe slightly lower correspondence between the model output and choices made in prospective cases.

Another limitation of this study is the inclusion of only a relatively small number of patients from a single center. As expected, the model based on the knowledge from the experts of the same center shows greater correspondence with the actual treatment of these patients than the nationwide model. The output of the nationwide model was higher for the patients who received CC, which shows that a higher percentage of experts nationwide would be inclined to perform surgery on these patients than the experts from the single center. This may be due to the fact that the relative importance of some variables is different between the 2 models, which means that UMCG experts value certain variables differently than the national group of experts do (e.g., gestational age and birth weight are considered relatively more important, cerebral ultrasound less). However, we also realized that the center-specific experts might be less inclined to perform LAP in general. To examine this thought, we went back to the results of the choice experiment^16^ and performed a chi-square test to learn if any differences exist between the inclination of the single center’s experts to perform surgery as compared with that of the other experts who took part in the choice experiment. Indeed, the analysis revealed that experts of the single center advised LAP relatively less often (53%) than the other experts (62%) did (*P* < 0.001). Combined, this may mean that UMCG experts consider factors such as birth weight and gestational age strong indicators for further quality of life, leaving them less predisposed to perform surgery if the values for these variables are weaker. To get a more accurate estimate of the nationwide model’s performance, the model should also be compared with a national database. This also allows testing its applicability on centers that, unlike the UMCG, offer the option of drainage as an intermediate solution. For this reason, the prospective study will consist of multiple centers in the Netherlands testing the nationwide model.

Still, this national model might not be as applicable internationally, given potential cultural differences with regard to the advice to perform LAP or offer CC. Similar to the differences we observed between the center-specific model and the nationwide model, we expect to see even larger differences between the national and an international model. For example, we may observe different choices related to extreme low gestational ages, as international differences exist in the lowest gestational ages at which preterm infants are being actively resuscitated after birth.^28^ Hence, to be able to use BAIT internationally, an international model should be developed. Again, this will help to increase transparency and study the differences, also between countries, in our decision making. We are currently performing choice experiments to build such an international model. In this model, we will actively seek representations from various countries to mitigate the potential for biased model outputs, taking into account that a model predominantly informed by the expertise of medium- to high-income countries might be less applicable in low-income countries. The development of this international model allows us to address another limitation of the current model, which is the imbalance within the expert panel, predominantly comprising medical experts rather than surgical ones. Our objective is to achieve a more balanced distribution among specialties, encompassing not only medical and surgical experts but, for example, also nurse practitioners and anesthesiologists.

With this study, we have aimed to investigate whether BAIT would be a suitable decision aid in the choice of LAP versus CC in surgical NEC. The challenge lies in the validation of these models, given that a true golden standard, whether based on short- or long-term outcomes, is not explicitly defined. This remains an ongoing challenge, for one, because determining the appropriate duration for these evaluations is complex. While some health issues may manifest by the time the infant is 2 y old, others might not become evident until later in childhood or even adulthood. In addition, measuring quality of life poses significant difficulties, further complicating the validation process. Hence, the most feasible approach was to compare the model output with observed choices. However, it is important to note that any discrepancies between the two do not necessarily reflect the model’s quality. In fact, one might even suggest that the differences found would require further analysis of the decision that was made. For example, we could look at long-term outcomes, including behavioral and neurodevelopmental outcome and quality of life of the LAP survivors. In future studies, it would be interesting to compare these long-term outcomes with the models’ output. The question remains if these are appropriate measures for the infant’s mental and physical health. In addition, it is often difficult to confirm if the correct choice has been made for patients who received CC and if the wrong choice is made for patients who died perioperatively or postoperatively. Hence, for now, we will begin by conducting evaluation studies to determine whether health care physicians consider the decision aid as currently constructed useful while also pinpointing the guidelines they require for using the model to enhance their decision-making processes.

The current study has shown a high correspondence between model output and actual decisions made in clinical care. Considering the limited dataset and possibly imperfect models, we still believe this shows BAIT is a promising decision aid for the decision to perform LAP or offer CC in severe NEC. It also shows which variables are considered important in the patient’s situation, aligning with doctors’ desires for such tools to be explanatory.^29^ In addition, the model can be updated continuously using real-life choices to guarantee its continuing relevance. In conclusion, the promising outcomes of this study, combined with our commitment to ongoing improvement and evaluation, suggest that BAIT has the potential to become a valuable decision aid in the NICU.
